# Interpretation of Absorption Bands in Airborne Hyperspectral Radiance Data

**DOI:** 10.3390/s90402907

**Published:** 2009-04-22

**Authors:** Karl H. Szekielda, Jeffrey H. Bowles, David B. Gillis, W. David Miller

**Affiliations:** 1 NRL/ASEE Summer Senior Faculty Fellow, on leave from the City University of New York, Hunter College, USA; E-Mail: szekielda@aol.com (K.H.S.); 2 Remote Sensing Division, Naval Research Laboratory, 4555 Overlook Ave, SW, Washington, DC 20375, USA; E-Mail: David.Gillis@nrl.navy.mil (D.B.G.); 3 Computational Physics Inc., Remote Sensing Division, Naval Research Laboratory, 4555 Overlook Ave, SW, Washington, DC 20375, USA; E-Mail: miller@cpi.com (W.D.M.)

**Keywords:** Photosynthetic pigments, hyperspectral remote sensing, fourth derivative, continuum removal, solar spectrum

## Abstract

It is demonstrated that hyperspectral imagery can be used, without atmospheric correction, to determine the presence of accessory phytoplankton pigments in coastal waters using derivative techniques. However, care must be taken not to confuse other absorptions for those caused by the presence of pigments. Atmospheric correction, usually the first step to making products from hyperspectral data, may not completely remove Fraunhofer lines and atmospheric absorption bands and these absorptions may interfere with identification of phytoplankton accessory pigments. Furthermore, the ability to resolve absorption bands depends on the spectral resolution of the spectrometer, which for a fixed spectral range also determines the number of observed bands. Based on this information, a study was undertaken to determine under what circumstances a hyperspectral sensor may determine the presence of pigments. As part of the study a hyperspectral imager was used to take high spectral resolution data over two different water masses. In order to avoid the problems associated with atmospheric correction this data was analyzed as radiance data without atmospheric correction. Here, the purpose was to identify spectral regions that might be diagnostic for photosynthetic pigments. Two well proven techniques were used to aid in absorption band recognition, the continuum removal of the spectra and the fourth derivative. The findings in this study suggest that interpretation of absorption bands in remote sensing data, whether atmospherically corrected or not, have to be carefully reviewed when they are interpreted in terms of photosynthetic pigments.

## 1. Introduction

Multi-spectral remote sensing in oceanic and coastal waters has proven to be a successful tool to interpret ocean color in terms of chlorophyll. However, with the present techniques, only limited information on other light harvesting pigments is available. Hyperspectral remote sensing of water-penetrating wavelengths (0.400 – 0.700 μm) can contribute significant information to the observation of many water column characteristics including depth, bottom type, and amounts of colored dissolved organic matter (CDOM), suspended sediments, and phytoplankton biomass. Additionally, the presence of phytoplankton accessory pigments can potentially be determined, providing information on the types and quantities of phytoplankton in the water. This knowledge can then be used to identify harmful algae, quantify biogeochemical cycles, and assess the trophic status of the ecosystem.

Similar to chlorophyll and carotenoids, phycobilins can be used as indicators for the presence of harmful algae blooms (HABs), i.e., the accessory pigments are taxonomically significant. Biliproteins such as phycoerythrin and phycocyanin have very distinct absorption features that are diagnostic for the presence of specific algae species. Red algae, for instance, contain phycoerythrin as the major pigment, whereas phycocyanin is the major and common component in blue-green algae, but allophycocyanin is nearly always a minor component [1].

Problematic in identification and quantification of pigments in coastal waters is the fact that chromophoric dissolved compounds (CDOM), inorganic particulate matter, organic debris and phytoplankton vary independently. Moreover, the independent variations of one class of material to another sets limits to the use of empirically and regionally developed algorithms for retrieving mass concentrations from optical data. It is shown later in this article that at high concentrations pigments other than chlorophyll can be detected by remote sensing [e.g. 2–6]. Furthermore, the spectral region where photosynthetic pigments absorb can be recognized by applying the fourth derivative techniques to spectra. Yet, results from identifying the spectral location of absorption bands may differ according to the sensor specifications, spectral band positions, spatial resolution, and sensitivity of the adapted processing scheme applied to the data [7].

Derivative analysis of spectra has been introduced as one promising technique for extracting absorption features from complex reflectance spectra in which they are not readily recognizable. However, identification of absorption peaks in derivative spectra depends on the magnitude of the absorption feature, width of the instrument’s spectral bands and spacing between them. For instance, two summed 20 nm-wide Gaussian bands that are separated by 14 nm at 543 and 557 nm, can be identified in the first derivative but can be better resolved with the fourth derivative, although a slight shift of 1 nm may occur [8]. In addition to the FWHM of, and spectral distance between, two absorption bands, unequal magnitudes and shapes of the absorption bands may lead to problems in their recognition.

A major uncertainty in interpreting radiance data with the fourth derivative is the impact of the atmosphere and the removal of its affects from the measured at-sensor-radiance. Standard atmospheric corrections applied to open ocean water (Case 1) often fail in the case of multi-component water (Case 2) because the assumption of a “dark pixel” (only atmospheric contributions to the measured radiance above ∼0.8 μm) is not valid. High radiance levels above 0.8 μm are frequently observed over dense plankton populations or waters with high sediment loads [5,7].

**Figure 1.**
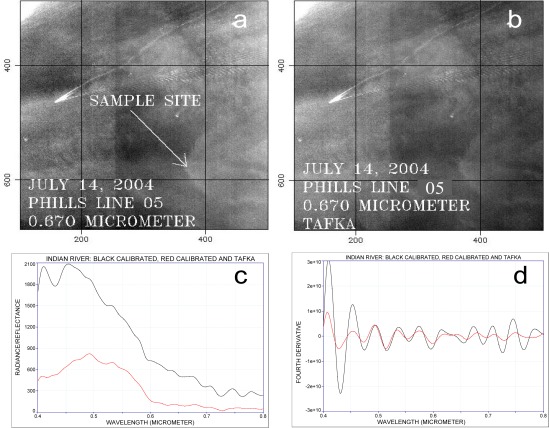
PHILLS data over the Indian River, FL. a: Calibrated data without atmospheric corrections. b: Calibrated and atmospherically corrected data. c: Radiance data (black) and atmospherically corrected reflectance data from sampling site in Figure a. d: Fourth derivative for spectra shown in Figure c.

Even under good conditions, performing atmospheric correction of remote sensing data may not completely remove Fraunhofer lines or atmospheric absorption bands. These absorbers may still be recognized in atmospherically corrected data. This is demonstrated in Figure 1 where data collected by the Portable Hyperspectral Imager for Low Light Spectroscopy (PHILLS) sensor, at a spectral resolution of 9.8 nm, and not atmospherically corrected were compared with the same data set after atmospheric corrections were applied. Fourth derivative analysis demonstrates that the same absorption bands can be detected in both scenes. This effect is likely the result of less than ideal conditions (such as clouds above the aircraft) because the modeled downwelling irradiance used in the calculation is not correct.

Based on these observations, a flight for acquisition of high resolution data was undertaken with the objective to analyze radiance data without applying atmospheric corrections and to identify spectral regions that might be diagnostic for photosynthetic pigments and were not contaminated by atmospheric signals. For this purpose, spectra that were acquired over oligotrophic and coastal water near Miami, FL were compared with the spectral response of a cyanobacteria bloom that was observed in the Potomac River south of Washington, DC. For absorption band recognition, the continuum removal of the spectra and the fourth derivative were applied to representative spectra from the scenes.

Absorption band recognition with the fourth derivative approach depends on spectral resolution, *Δλ*, of the instrument. The magnitude of spectral derivatives is depressed with increasing *Δλ* – and thus absorption bands are not detectable if *Δλ* is large enough [9,10]. For instance, the chlorophyll absorption bands at spectral resolutions from 1 to 4 nm spectra were similar in the fourth derivatives whereas at 5 nm resolution, the chlorophyll *a* peak began to deteriorate and was less pronounced at 7 nm resolution [11]. This observation has to be taken into account when radiance data without, and even with, atmospheric corrections are intended for pigment identification because atmospheric absorption bands and Fraunhofer lines appear close to those of photosynthetic pigments and they can merge. Thus, an apparent detection of a pigment may in fact be a residual atmospheric absorption line. In addition, pigments absorption peaks occur all across the visible spectrum as demonstrated in Table 1. Although the table is not complete, it demonstrates that some pigments may not be independently recognized by their spectral absorption bands at a 5 nm or larger spectral resolution.

As the spectral resolution of remote sensing data is critical for identifying the precise spectral location of absorbers, the merging and shifting of spectral bands in response to varying spectral resolutions also needs to be taken into account. The following interpretation of spectra should be considered as qualitative with no attempt to quantify the amount of the spectral absorbers, i.e., the main objective here is the determination of the presence and location of particular pigments and not the amount of the pigments present. In the following sections, we will address those aspects in the interpretation.

## 2. Data and Methods

### 2.1. Remote Sensing Data Acquisition

Remote sensing data were acquired with the two instruments, the Compact Airborne Spectrographic Imager (CASI) and PHILLS (More details about PHILLS’ design, its SNR, characterization and calibration can be found in Davis *et al*. [21]) Both instruments are pushbroom imagers that record a scene by the successive build-up of individual lines (a spatial line typically crosstrack from the direction of aircraft motion) of data. Each line has spatial information recorded along one dimension of a focal plane array while spectral information is recorded along the other.

For this particular experiment, the spectral resolution of CASI was set at 2.4 nm. The spectral range examined was between 0.4 μm and 0.7 μm where most photosynthetic pigments show absorption. This range also covers the location of the major Fraunhofer lines (in particular at 0.431 μm) and the oxygen absorption band at 0.687 μm; both were used as references for testing the spectral accuracy of the measurements. The data were not atmospherically corrected and it was assumed that during the short flight no major atmospheric changes took place, an assumption that may not be necessarily accurate.

CASI data showed noise that was partly suppressed with an enhanced Lee filter by sub-setting spectrally the CASI bands 12 (0.3988 μm) to 165 (0.7601 μm) with a filter size 3 and a damping factor of 1. Output data were further processed with ENVI (ITT, Boulder, CO) and TableCurve (Aspire Software, Ashburn, VA) software packages. To further improve the signal to noise ratio in the data, the collected spectra were locally averaged. PHILLS data was collected in the Potomac River over a plankton bloom on July 12, 2005. The final data used in this analysis had a spectral resolution of 5 nm.

### 2.2. Use of the Fourth Derivative

The usefulness of applying the fourth derivative on absorption band recognition in spectra has been documented in numerous studies [2,3,8,11]. This approach was selected because of its sensitivity in detecting spectral heterogeneity under the assumption of noise-free data sets, although the analysis still depends on parameters set by the operator. In order to keep the subjective aspects to a minimum, the algorithms for obtaining the fourth derivative were kept constant. As the selection of the windows for application of the smoothing filter and the passes is a subjective method, the minimum requirements for smoothing the fourth derivative were kept constant. They were specified with a one-sided moving window of three, and passes with a number of sequential applications of the smoothing filter was also kept at three. This procedure was equally executed for all data subjected to the fourth derivate. Final results of the fourth derivative were smoothed using the cubic option in TableCurve.

### 2.3. Continuum Removal

The removal of the continuum allows normalization of spectra when comparing individual absorption features from a common baseline. It considers the continuum onto which individual absorption bands are superimposed and absorption features that are superimposed on the wing of larger absorption features. In ENVI, the continuum is considered as the convex hull fit over the top of a spectrum using straight-line segments that connect local spectra maxima (another way to think about it is as the largest possible number of lines connecting points on the spectrum for which no points are below the connected lines). Basically, the continuum is removed by dividing it into the actual spectrum for each pixel in the image. The resulting spectrum, and finally the continuum-removed image, are equal to 1.0 where the continuum and spectra match, and are less than 1.0 where absorption features occur.

## 3. Results and Discussion

### 3.1. The Effect of Spectral Resolution on Absorption Band Recognition

**Figure 2.**
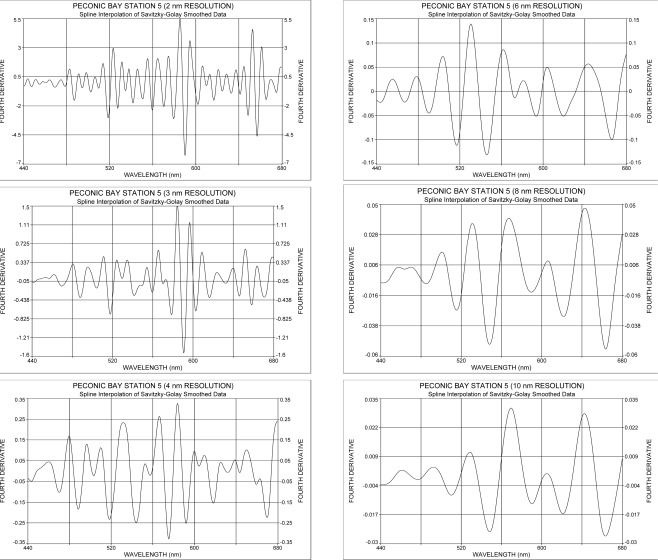
Simulation of spectral resolution for water column upwelling irradiance based on actual measurements at 1.7 nm resolution 10 cm below the surface.

Recognition of absorption bands and their possible merging depend on the spectral resolution of the spectrometer, a fact that can be visualized by simulating various spectral resolutions as shown in Figure 2. A spectrum, originally measured at a spectral resolution of 1.7 nm, was degraded to resolutions of 2, 3, 4, 6, 8 and 10 nm and then subjected to the fourth derivative. The results show that in response to spectral resolution changes the absorption bands change in amplitude, and finer absorption bands either merge into the larger ones or are lost completely. To reduce the possibility of pigment absorption peaks merging together at higher spectral resolutions (> 5 nm), an attempt was made to reveal pigments at a resolution of 2.4 nm by comparing spectra from highly eutrophicated water with those obtained from oligotrophic and coastal waters.

### 3.2. Spectra over Coastal Waters and the Florida Current

**Figure 3.**
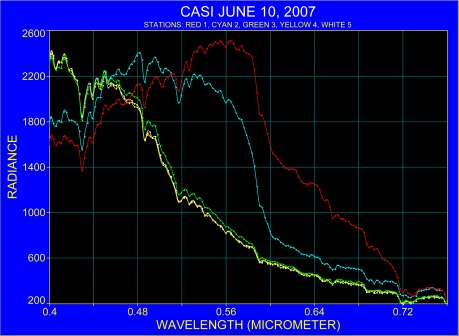
Averaged air-borne radiance spectra expressed in digital numbers.

In order to collect spectra over both coastal and oligotrophic waters in a single scene, the CASI instrument was flown from the Florida coast out into the Florida Current. Averaged spectra derived from the overflight on June 10, 2007 are displayed in Figure 3. Two representative regions were examined in detail. The first (station 2) was located in nearshore waters where plume structure was observed in the imagery and is an average of 73 individual spectra. The second (station 5) is within the clear waters of the Florida Current and the spectrum used from that area and is the average of 48 spectra. The main difference of the two spectra lies in the higher radiance in the near-shore water between approximately 0.44 μm and 0.60 μm. The difference in radiances between station 2 and station 5 reveals higher absorption in the coastal water for the spectral region between 0.40 μm and 0.47 μm, whereas starting at around 0.47 μm the near shore station is characterized by elevated radiance.

### 3.3. Estimates of Accuracy of Absorption Band Locations in CASI Data

Identification of absorption bands requires precise wavelength knowledge. Therefore, an estimate was made for the spectroscopic precision by comparing known location of absorption lines in actual spectra and after applying the fourth derivative. Common to all spectra, are two major absorption bands one of which is the result of the merged Fraunhofer lines near 0.431 μm and other is the atmospheric oxygen absorption band at 0.687 μm. To estimate the accuracy of the spectra, the position of the Fraunhofer line and the atmospheric oxygen absorption band were used for all stations that are shown in Figure 3. These exercises showed that the spectral location of absorbers can be recognized within one nanometer and that no significant shift in absorption bands can be detected between the original spectra and the fourth derivative of the spectra.

**Figure 4.**
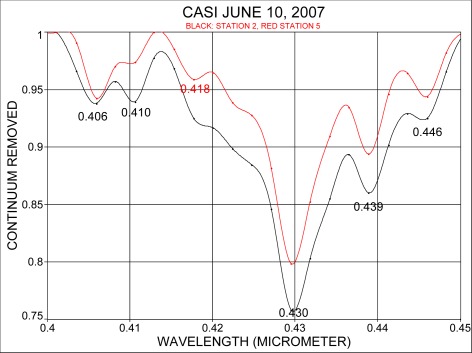
Continuum removal for the spectral range 0.40 to 0.45 μm at 2.4 nm resolution.

**Figure 5.**
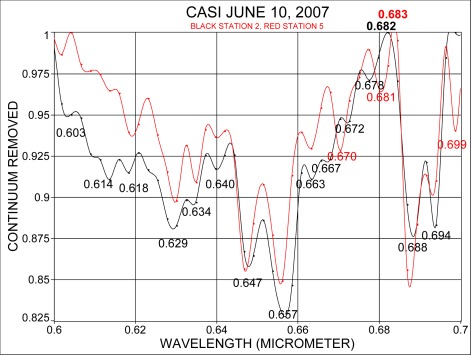
Continuum removal for the spectral range 0.60 μm to 0.70 μm at 2.4 nm resolution.

Spectra from stations 2 and 5 were also analyzed by removing the continuum. Figure 4 shows the spectral region between 0.40 μm and 0.45 μm, and Figure 5 considers the spectral range from 0.60 μm to 0.70 μm. Figure 4 shows that the Fraunhofer line at 0.430 μm is clearly resolved and in addition, the band locations at 0.439 μm and 0.446 μm, in the vicinity of chlorophyll and carotenoids, absorption are recognized. The range 0.60 μm to 0.70 μm (Figure 5) shows that the oxygen absorption band is recognized for both water masses at 0.688 μm. An indication for the α-chlorophyll absorption band is apparent at 0.667 μm for the coastal station and at 0.670 μm for the Florida Current. The spectral position where normally fluorescence due to chlorophyll occurs was found in spectra at 0.682 μm for the near shore water and at 0.683 μm for the Florida Current.

### 3.4. Interpretation of Derivative Spectra at 2.4 nm Resolution

**Figure 6.**
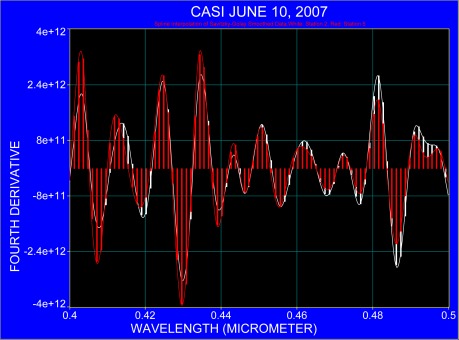
The fourth derivative applied to the averaged spectra at station 2 (white) and station 5 (red) for the spectral region 0.4 μm to 0.5 μm.

**Figure 7.**
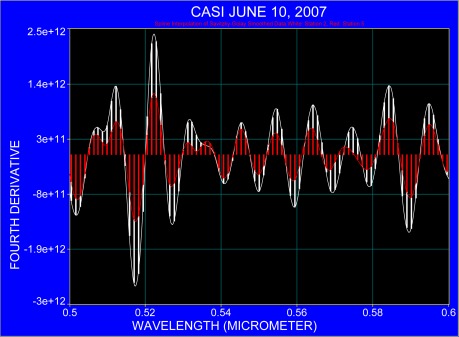
The fourth derivative applied to the averaged spectra at station 2 (white) and station 5 (red) for the spectral region 0.5 μm to 0.6 μm.

**Figure 8.**
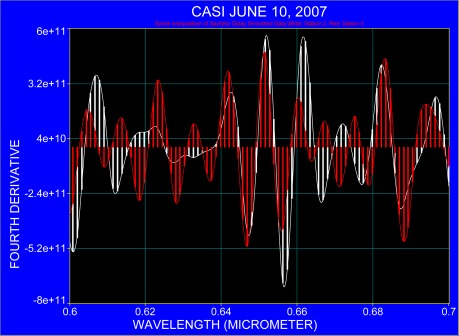
The fourth derivative applied to the averaged spectra at stations 2 (white) and station 5 (red) for the spectral region 0.6 μm to 0.7 μm.

The following figures compare the fourth derivative for the nearshore station 2 and offshore station 5, taking into account the region of pigment absorption as listed in Table 1. For clarity, the fourth derivative spectra ranging from 0.40 μm to 0.70 μm are presented in three sections in Figures 6 to 8 where each spectral section covers an interval of 0.1 μm. The spectra were processed with the same running window and only the y-axis was graphically adjusted. Both spectra demonstrate that atmospheric absorption and Fraunhofer lines coincide with the spectral region where pigment absorption occurs in the blue part of the spectrum (Figure 6). The possible location of pigment absorption is indicated at some wavelengths where a slight spectral shift of around 3 nm is observed (Figure 7). The most rational explanation is that the absorption bands differ in their full width at half maximum (also indicated in spectra after continuum removal; compare with Figures 4 and 5) and that their different amplitudes result in varying steepness which may initiate merging of pigment absorption and other absorption bands.

Figure 8 covers the spectral range between 0.6 μm to 0.7 μm where coastal water shows two apparent additional absorption bands at 0.612 μm and 0.666 μm; the latter is an indicator for the absorption of chlorophyll while the radiance maximum at 0.683 μm can be associated with sun-induced fluorescence [22–24].

### 3.5. Spectral Interpretation of a Cyanobacteria Bloom Conditions with Reference to Case 2 Water

The above conclusion might be valid only for a certain biomass concentration range that is normally expected in the investigated coastal water and in the Florida Current. To elaborate further on the detection of absorption bands at high phytoplankton concentrations, a cyanobacteria bloom observed in the Potomac River with PHILLS was compared with the spectrum obtained over the Florida Current.

During the flight over the Potomac River with PHILLS on July 12, 2005, limited ground measurements were available. Cell counts two days after the overflight showed that plankton assemblages in the region of the overflight were dominated by about 60% to 75% cyanobacteria. Blooming of *Microcystis spp.* persisted throughout the month of July (personal communication T. Donato) and visual observations from ship cruises during the month of July identified surface blooms in the form of foamy brownish/green surface aggregates and green “flocs”. Because a cyanobacteria bloom was observed, chlorophyll *a*, zeaxanthin, and biliproteins in the form of allophycocyanin, phycocyanin and phycoerythrin were expected as the major photosynthetic pigments that would affect the radiance data.

**Figure 9.**
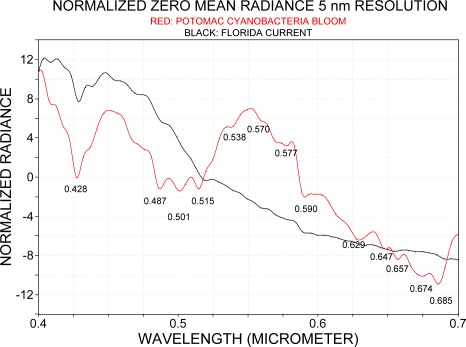
Comparison of zero mean average spectra obtained over the Florida Current on June 10, 2007, and a cyanobacteria bloom in the Potomac River on July 12, 2005. Both spectra are shown at 5 nm spectral resolution. Original units for the Florida Current are in digital numbers and for the Potomac River data were in W m^−2^ sr^−1^ μm^−1^.

**Figure 10.**
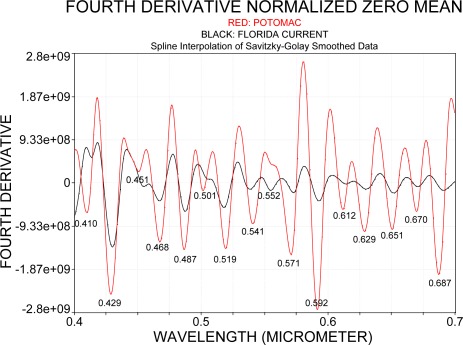
Fourth derivative of mean zero normalized spectra obtained over the Florida Current and a bloom in the Potomac (refer to Figure 9), both at 5 nm spectral resolution.

As PHILLS data were obtained at a spectral resolution of 5 nm, the spectra from the Florida Current that were originally recorded at 2.4 nm resolution were degraded to a resolution of 5 nm and both spectra were normalized to their zero mean average. This procedure partly compensates for different illuminations since the observations were taken on different dates. The corresponding spectra are shown in Figure 9. The spectrum from the Florida Current has a rather smooth radiance spectrum compared to that of cyanobacteria bloom in the Potomac River. At shorter wavelengths, between 0.45 μm and 0.52 μm, three strong absorption bands were identified in the cyanobacteria blooms which, except for the strong Fraunhofer line at 0.428 μm, are almost indistinguishable in the Florida Current spectrum. The cyanobacteria bloom in the Potomac River further showed a pronounced reflection maximum at around 0.55 μm. In the range 0.55 μm to 0.7 μm increasing water absorption towards longer wavelengths was responsible for decreasing radiance, but several absorption bands were recognized in the radiance spectrum of cyanobacteria. For more precise band locations and comparison of both spectra, the fourth derivative was applied to the spectra of which results are presented in Figure 10.

The two spectra are described in connection with Table 1 in which the spectral absorption bands of pigments are listed. This comparison serves to demonstrate that spectral absorption of pigments coincides with the bands that can be identified both in the Florida Current and in the bloom in the Potomac without claiming that pigments were the cause for absorption as detected with the fourth derivative, and pointing out rather where absorption of photosynthetic absorption bands should or could occur.

The absorption bands recognized with the fourth derivative fall together with the spectral bands where phytoplankton pigment absorption can be expected: at 0.410 μm for chlorophyll *a*, 0.451 μm for chlorophyll *c*; 0.468 μm for chlorophyll c_1_, 0.487 μm for zeoxanthin, 0.501 μm for phycoerythrin, 0.541 μm for phycoerythrin, 0.552 μm for phycocyanin, 0.571 μm for phycoerythrin, 0.592 μm for overlapping absorption of phycoerythrin and chlorophyll *c*; 0.612 μm and 0.629 μm for phycocyanin, 0.651 μm for allophycocyanin, 0.670 μm for chlorophyll; and 0.687 μm shows the oxygen absorption band.

**Figure 11.**
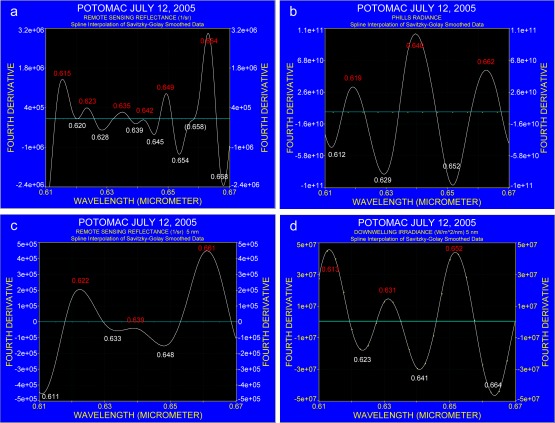
Fourth derivative spectra of a cyanobacteria bloom in the Potomac River on July 12, 2005. Numbers in white indicate the spectral location of a trough and numbers in red the spectral location of a crest. a: Remote sensing reflectance (original data expressed as R_rs_ in sr^−1^) taken aboard a research vessel at spectral resolution of 1.4 nm. b: Radiance spectrum is measured with PHILLS at 5 nm resolution (original data expressed in W m^−2^ sr^−1^ μm^−1^). c: Spectrum shown in Figure 11a degraded to a spectral resolution of 5 nm. d: Downwelling irradiance E_d_ (original data expressed in W m^−2^ nm^−1^). Note that the magnitude of the y-axis varies in the different figures.

As the cyanobacteria bloom should indicate phycocyanin absorption, the spectral region between 0.61 μm and 0.67 μm was further analyzed. In the literature, the in vivo phycocyanin absorption of cyanobacteria was reported by Dekker *et al*. [25] at 0.627 μm and at around 0.615 μm by Simis *et al*. [26,27]. Kutser *et al*. [6] determined that the phycocyanin absorption in reflectance data was strongest at about 0.615 μm. The spectral region where phycocyanin absorption should occur in radiance data was compared with ground observations that were collected during the overflight with PHILLS. Figures 11a and 11b compare the fourth derivative for the remote sensing reflectance on the ground with the fourth derivative of the radiance spectrum that was collected with PHILLS. At spectral resolution of 1.4 nm (Figure 11a) the ground-based spectrometer resolved absorption of cyanobacteria in the spectral range from 0.61 to 0.67 μm, with absorption bands at 0.628 μm and 0.654 μm. PHILLS data that are recorded at a resolution of 5 nm as shown in Figure 11b shows bands at 0.629 μm and 0.652 μm. In order to compare better PHILLS and ground truth data, the latter was degraded to a spectral resolution of 5 nm and is shown in Figure 11c. The degraded ground spectrum reveals absorption bands at 0.633 μm and 0.648 μm but indicates a slight shift in the band position of about 5 nm when compared with the results presented in Figure 11a.

Considering that two different methods were used to identify the absorption bands and that PHILLS data were not atmospherically corrected, it is evident that both PHILLS and the ground-based spectra concur in the spectral region where phycocyanin absorption could be identified. On the other hand, the fourth derivative for remote sensing reflectance suggests that atmospheric absorbers and Fraunhofer lines still contribute to the spectrum. This is apparent in the fourth derivative of E_d_, shown in Figure 11d, which does not exhibit absorption that could be attributed to the wavelengths where cyanobacteria absorb.

**Figure 12.**
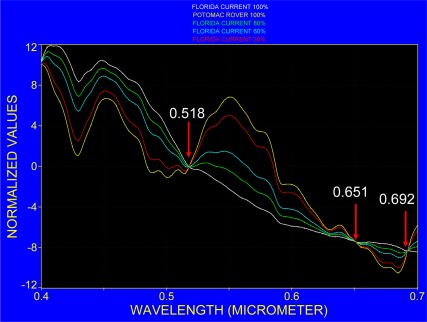
Normalized zero mean spectra for the Florida Current and the Potomac River (compare with Figure 9) and mixture of spectra at three different proportions.

**Figure 13.**
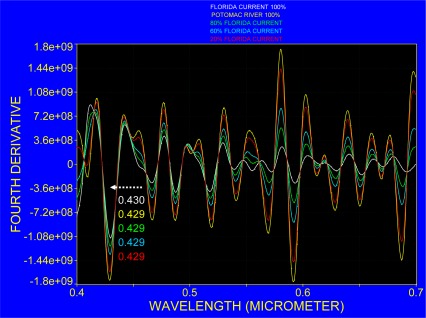
Fourth derivative of the spectra shown in Figure 12.

The recognition of phycocyanin is based on the high cell density in the cyanobacteria bloom that may build surface scum with a spectral response that resembles that of vegetation. In order to demonstrate the possible spectral change with respect to changing cell concentrations, the normalized spectra shown in Figure 9 were further processed to simulate a concentration gradient from coastal water (Potomac River) to Case 1 water (Florida Current) by mixing their spectra at different proportions. The resulting spectra and their fourth derivative are presented in Figures 12 and 13. Although this procedure assumes linear mixing between two water masses and ignores any physical and chemical changes during the mixing process, it highlights the fact that at certain parts of the spectrum, the absorption bands interact and introduce a spectral shift, change in slope and show variation in their amplitudes. This is visible in particular at around 0.46, 0.5, 0.55 and 0.62 μm.

**Figure 14.**
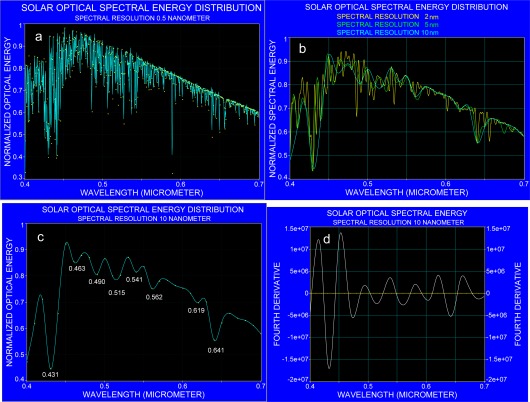
Analysis of the solar spectrum. a: spectral resolution at 0.5 nm; b: spectral resolution of the solar spectrum at 2, 5 and 10 nm respectively; c: the solar spectrum at 10 nm resolution repeated separately for clarity; d: fourth derivative of the solar spectrum at 10 nm spectral resolution.

The similarity of the fourth derivative of all spectra indicates that the absorption bands, found with the fourth derivative of radiance data, originate either from the atmosphere and/or the solar spectral lines. In order to support this observation, a solar spectrum [28] was degraded to various spectral resolutions and subjected to the fourth derivative. The results are shown in Figure 14 that represent the solar spectrum at 0.5, 2, 5 and 10 nm, respectively, and the fourth derivative spectra for the solar spectrum at 10 nm spectral resolution. By comparing Figure 14a with that of 14b, it is evident that the strong absorption band at around 0.430 μm, can be recognized in radiance data obtained with a spectral resolution of 2.4 nm (see Figure 4), as a result of merging Fraunhofer lines.

### 3.6. Summary and Conclusions

Through analysis of the spectral region from 0.4 to 07 μm oligotrophic water and near coastal water show similar spectral responses with respect to the locations of absorbers. However, the amplitude of spectra shows a deepening of the absorption bands for the near coastal water compared to spectra that were collected over oligotrophic waters. For the spectral range of 0.6 to 0.7 μm, additional absorbers can be identified in near-coastal water at 0.667, 0.672 and 0.678 μm although spectral shift in the absorption bands was noticed. The fourth derivatives of spectra for oligotrophic water (Florida Current) and the near coastal water have almost identical spectra between 0.4 and 0.5 μm. The region between 0.5 and 0.6 μm also shows similar absorption bands for both water masses, except that the amplitude changes for the near coastal water. An increase in amplitude and a shift in band location are observed in the spectral region between 0.6 and 0.7 μm where absorption bands at around 0.612 and 0.666 μm in the near coastal water are well pronounced.

A comparison of the spectrum of a cyanobacteria bloom in the Potomac River with the spectrum of the Florida Current at spectral resolution of 5 nm revealed that some pigments at high concentrations may be recognized in radiance spectra. The fourth derivative of the spectra for the three water masses that were considered in this study show that the fourth derivative of radiance spectra extracts to a high degree atmospheric absorbers and Fraunhofer lines. This has been confirmed with the fourth derivative of the solar spectrum that was analyzed at various spectral resolutions. Although the Fraunhofer lines are very narrow spectral lines, they merge if measurements are made over a wide band width. The spectral proximity of Fraunhofer lines and the location of absorption of individual pigments in the neighborhood of a few nanometers away further complicate the interpretation of radiance spectra even at a spectral resolution of 2.4 nm. Therefore, measurements at a higher spectral resolution might be useful to separate the Fraunhofer lines in radiance measurements and this could lead to better identification of broader absorption bands of photosynthetic pigments in the continuum. Such an approach might be useful especially in highly eutrophicated and near-coastal regions. Furthermore, the findings in this study imply that interpretation of absorption bands in radiance data, whether atmospherically corrected or not, have to be carefully reviewed when they are interpreted in terms of photosynthetic pigments.

## Figures and Tables

**Table 1.** Absorption maxima of biliproteins, carotenoids and chlorophylls. Superscripts indicate reference number.

**Pigment**	**Absorption_MAX_ (μm)**	**Pigment**	**Absorption_MAX_ (μm)**
Chlorophyll a	0.441^16)^	B-Phycoerythrin	0.565^18)^
Chlorophyll c	0.454^20)^	Phycoerythrobilin (PE)	0.565^14)^
Carotenoids	0.462^15)^	R-Phycoerythrin (RPE)	0.566^A)^
Chlorophyll c_1_	0.466^17)^		0.566^B)^
Diadinoxanthin			0.568^1)^
Chlorophyll *b*	0.470^20)^	R-Phycoerythrin I	0.568^18)^
	0.470^15)^	Phycoerythrin maxima	0.570–0.575^16)^
	0.471^16)^	Phycoerythrocyanin	0.575^A)^
Zeaxanthin	0.488^20)^	Chlorophyll c	0.589^17)^
Fucoxanthin	0.490^15)^		0.588^20)^
19’-acylfucoxanthin, Diadinoxanthin	0.490^19)^	R-Phycoerythrin	0.598^1)^
		C-Phycocyanin	0.615^A)^
Phycoerythrin rich in PUB	0.492^15)^		0.615^1)^ 0.616^B)^
	0.495^17)^		0.615^18)^
Phycourobilin (PUB)	0.495^14)^	R-Phycocyanin	0.615^18)^
R-Phycoerythrin	0.495^A)^	Phycocyanin	0.630^6)^
	0.498^18)^		0.630^1)^
PE maxima	0.499^16)^	R-Phycocyanin	0.630^16)^
Fucoxanthin	0.515^20)^	Chlorophyll c	0.635^20)^
Fucoxanthin	0.535^20)^	Chlorophyll *c_1_*,	
Phycoerythrin	0.538^16)^	Fucoxanthin,	0.639^17)^
	0.540^1)^	19’-acylofucoxanthin	
	0.540^18)^	Chlorophyll *b*	0.652^15)^
	0.541^1)^	Cyanobacteria	0.650^13)^
	0.544^1)^	Allophycocyanin	0.652^A)^
	0.545^A)^		0.650^1)^
	0.545^1)^		0.652^B)^
B-Phycoerythrin (BPE)	0.545^A)^	Chlorophyll^)^	0.671^12)^
	0.546^B)^		0. 675^13)^
R-Phycocyanin	0.553^18)^		0.676^1)^
	0.555^A)^	Chlorophyll *a*	0.674^15)^
Fucoxanthin	0.554^20)^		0.683^16)^
C-Phycoerythrin	0.563^18)^		0.677^17)^
R-Phycoerythrin II	0.564^18)^		0.675^20)^

Notes:

A) Phycobiliproteins ProZyme www.prozyme.com/technical/pbvrwdata.html,

15) adjusted for wavelength shift in solution except for phycoerythrin rich in PEB (phycoerythrobilin) and rich in PUB (phycourobilin),

16) data for Rhodophyta,

B) Cyanotech Corporation www.phycobiliprotein.com,

20) adjusted peaks.

## References

[1] Kirk J.T.O. (1994). Light and Photosynthesis in Aquatic Ecosystems.

[2] Andréfouet S., Hochberg C., Che L.M., Atkinson M.J. (2003). Airborne hyperspectral detection of microbial mat pigmentation in Rangiroa atoll (French Polynesia). Limnol. Oceanogr.

[3] Richardson L.L., Buisson D., Liu C.J., Ambrosia V. (1994). The detection of algal photosynthetic accessory pigments using airborne visible-infrared imaging spectrometer (AVIRIS) spectral data. Mar. Technol. Soc. J.

[4] Quibell G. (1992). Estimating chlorophyll concentrations using upwelling radiance for freshwater algal genera. Int. J. Remote Sens.

[5] Kutser T. (2004). Quantitative detection of chlorophyll in cyanobacterial blooms by satellite remote sensing. Limnol. Oceanogr.

[6] Kutser T., Metsamaa L., Strömbeck N., Vahtmäe E. (2006). Monitoring cyanobacterial blooms by satellite remote sensing. Estuar. Coast. Shelf Sci.

[7] Reinart A, Kutser T. (2006). Comparison of different satellite sensors in detecting cyanobacterial bloom events in the Baltic Sea. Remote Sens. Environ.

[8] Butler W.L., Hopkins D.W. (1970). Higher derivative analysis of complex absorption spectra. Photochem. Photobiol.

[9] Tsai T., Philpot W. (1998). Derivative analysis of hyperspectral data. Remote Sens. Environ.

[10] Torrecilla E., Aymerich I.F., Pons S., Piera J. (2007). Effect of spectral resolution in hyperspectral data analysis. IEEE Int. Geosci. Remote Sens. Symp.

[11] Roelke D.L., Kennedy C.D., Weideman A.D. (1999). Use of discriminant and fourth-derivative analyses with high-resolution absorption spectra for phytoplankton research: Limitations at varied signal-to-noise ratio and spectral resolution. Gul. Mex. Sci.

[12] Szekielda K.H., Gobler C., Moshary F., Gross B., Ahmed S. (2003). Spectral reflectance measurements of estuarine waters. Ocean Dyn.

[13] Garver S.A., Siegel D.A. (1994). Variability in near-surface particulate absorption spectra: What can a satellite ocean color imager see?. Limnol. Oceanogr.

[14] Hoge F., Wright C.W., Lyon P.E., Swift R.N., Yungel J.K. (1999). Satellite retrieval of the absorption coefficient of phytoplankton phycoerythrin pigment: Theory and feasibility status. Appl. Opt.

[15] Bidigare R.R., Ondrusek M.E., Morrow J.H., Kiefer D.A. (1990). *In vivo* absorption properties of algal pigments. Proc. SPIE.

[16] Smith C.M., Alberte R.S. (1994). Characterization of *in vivo* absorption features of chlorophyte, phaeophyte and rhodophyte algal species. Mar. Biol.

[17] Millie D.F., Kirkpatrick G.J., Vinyard B.T. (1995). Relating photosynthetic pigments and in vivo optical density spectra to irradiance for the Florida red-tide dinoflagellate *Gymnodinium Breve*. Mar. Ecol. Prog. Ser.

[18] Carra P.O. (1965). Purification and N-terminal analysis of algal biliproteins. Biochem. J.

[19] Johnsen G., Samset O., Granskog L., Sakshaug E. (1994). *In vivo* absorption characteristics in 10 classes of bloom-forming phytoplankton: taxonomic characteristics and responses to photoadaptation by means of discriminant and HPLC analysis. Mar. Ecol. Prog. Ser.

[20] Aguirre-Gomez R., Weeks A.R., Boxall S.R. (2001). The identification of phytoplankton pigments from absorption spectra. Int. J. Remote Sens.

[21] Davis C.O., J. Bowles J., Leathers R.A., Korwan D., Downes T., Snyder W.A., Rhea W.J., Chen W., Fisher J., Bissett W.P. (2002). Ocean PHILLS hyperspectral imager: design, characterization, and calibration. Opt. Express.

[22] Hoge F., Swift R. (1987). Ocean color spectral variability studies using solar induced chlorophyll fluorescence. Appl. Opt.

[23] Gower J.F.R., Doerfer R., Borstad G.A. (1999). Interpretation of the 685 nm peak in water-leaving radiance spectra in terms of fluorescence, absorption and scattering, and its observation by MERIS. Int. J. Remote Sens.

[24] Gitelson A. (1992). The peak near 700 nm on radiance spectra of algae and water: relationship of its magnitude and position with chlorophyll concentration. Int. J. Remote Sens.

[25] Dekker A.G., Malthus T.J., Goddijn L.M. Monitoring cyanobacteria in eutrophic waters using airborne imaging spectroscopy and multispectral remote sensing systems.

[26] Simis G.H., Tijden M., Hoogveld H.L., Gons H.J. (2005). Optical changes associated with cyanobacteria bloom termination by viral lysis. J. Plankton Res.

[27] Simis G.H., A. Ruiz-Verdú A., Dominguez-Gomez J.A., Pena-Martines R., Peters S.W.M., Gons H.J. (2007). Influence of phytoplankton pigment composition on remote sensing of cyanobacterial biomass. Remote Sens. Environ.

[28] Lobel A. (2008). SpectroWeb: oscillator strength measurements of atomic absorption lines in the Sun and Procyon. J. Phys. Conf. Ser.

